# New Technique for Wedge Selection in Direct Class II Restorations: A Pilot Study

**DOI:** 10.3390/jcm13051324

**Published:** 2024-02-26

**Authors:** Tania Gancedo-Gancedo, Benjamín Martín-Biedma, Javier Domínguez-Cachón, Sara Garrido-Parada, Victoria Ababii, Patricia Pereira-Lores, Sandra García-Varela, Pablo Castelo-Baz

**Affiliations:** 1 Endodontics and Restorative Dentistry Unit, School of Medicine and Dentistry, University of Santiago de Compostela, 15705 Santiago de Compostela, Spain; javicachon99@gmail.com (J.D.-C.); saragarridodes@gmail.com (S.G.-P.); patriciapereiralores@gmail.com (P.P.-L.); sangava17@gmail.com (S.G.-V.); 2 Oral Sciences Research Group, Endodontics and Restorative Dentistry Unit, School of Medicine and Dentistry, Health Research Institute of Santiago de Compostela (IDIS), University of Santiago de Compostela, 15705 Santiago de Compostela, Spain; b.martinbiedma@gmail.com (B.M.-B.); pablocastelobaz@hotmail.com (P.C.-B.); 3 Department of Odontology, Periodontology and Oral Pathology, Nicolae Testemițanu State University of Medicine and Pharmacy, 2004 Chișinău, Moldova; victoria.ababii@usmf.md

**Keywords:** direct restoration, class II, composite resins, matrix, wedge

## Abstract

**Background:** Performing an appropriate Class II direct restoration is a great challenge. The correct selection of the matrix system and the elements used for its stabilization will have a great impact on the result. The aim of this study is to show a new digital method for a predictable selection of the wedge and compare it with the conventional method. **Methods:** Sixty patients were randomly divided into two groups. In Group 0, the wedge was selected intraoperatively by visual examination, while in Group 1 the wedge was selected preoperatively through the measurement of the cervical embrasure on the bite-wing radiography. The number of wedges used, modifications to them, and tissue damage were registered, along with the quality of the proximal contact and the marginal adaptation. **Results:** Student’s *t*-test revealed a statistical difference between the number of wedges used, which was higher in Group 0 (*p* < 0.05). Pearson Chi-square test showed that tissue damage was statistically higher in Group 0 (*p* < 0.05), while there was no statistically significant difference between groups in wedge modifications (*p* > 0.05). Group 1 revealed a higher frequency of satisfactory proximal contact and marginal adaptation (*p* < 0.05). **Conclusions:** This new technique reduces wedges waste and tissue damage and provides an adequate interproximal anatomy.

## 1. Introduction

Class II cavities are highly prevalent in daily clinical practice, and their rehabilitation can be a challenge for the clinician [1,2]. According to a retrospective study with 18.5 years of observation, it was reported that Class II restorations had a higher failure rate than Class I restorations, this difference being statistically significant [3]. Within the first five years, primary issues with direct restorations in posterior teeth commonly include restoration fractures, secondary caries, and marginal gaps [4,5].

Reproducing the original interdental anatomy and obtaining a good marginal adaptation are key factors to reduce the failure rate [1,6,7]. What is required is the presence of a tight, anatomical contact area that prevents food impaction, recurrent caries, periodontal disease, and tooth movement, among others [1,8].

In order to restore this area, its anatomy must be known. The natural contact zone has an area of approximately 1.5–2 mm [9]. In the premolar/molar region, from an occlusal view, this area is located at the junction between the middle and the buccal third in a bucco-lingual direction. From a buccal perspective, the interproximal area aligns with the maximum contour of the approximal surface; when observed cervico-occlusally, the contact area is positioned at the transition between the middle and the occlusal third [10,11].

The correct selection of the matrix system, as well as the elements used to achieve its stabilization and the required interdental separation, are essential for acquiring the aforementioned objectives [12,13]. Although all available matrices have deficiencies in reproducing the interproximal contour, several studies concluded that pre-contoured sectional matrices are the best option [1,12,14]; despite the knowledge gap that exists regarding the development of protocols for performing Class II restorations and, in particular, to replicate the interproximal anatomy [7], details regarding the size and thickness of some of these matrices, and the criteria for their selection, have been provided recently [7,14].

Sectional matrices are often used with a separation clamp to achieve an interdental separation that compensates the thickness of the matrix and provide coronal stability [7,14], and a wedge, which also exerts an interdental separation effect in addition to adapt the matrix to the cervical contour and provide stability [7,14,15,16]. If the wedge is too narrow, the matrix will not adapt properly to the tooth surface and a correct marginal seal will not be obtained [7,15]. The presence of overhanging restorations has been correlated with a higher severity of periodontal disease [17,18]. In a recent study, bone loss over 25 years in patients with overhanging restorations was evaluated; bone loss adjacent to over contoured restorations was a mean of 0.16 mm/year, while in adjacent unrestored teeth it was a mean of 0.06 mm/year [19]. On the other hand, a wedge that is too wide will cause the matrix to exceed the space determined for the restoration, causing a concavity [7,15]. A correct height is also necessary in order not to compromise the interproximal space; a high wedge can lead to an occlusally displaced contact point, while a low one can cause a gingivally misplaced contact area and compressed interdental papilla [7,15]. Although this is known, there are no protocols to objectively determine the appropriate size of the wedge, which is selected intraoperatively according to the clinician’s criteria [7,12], with the possible loss of time and material and increased risk of tissue damage and procedural errors.

The aim of this study is to evaluate the most effective method for wedge selection in direct Class II restorations, comparing the conventional technique where the wedge was selected visually intraoperatively with a new digital technique, where measurements were taken from the preoperative bite-wing radiography to determine wedge size.

## 2. Materials and Methods

### 2.1. Sample Size Calculation

The sample size was based on a previous research study that provided a formula for its calculation in pilot studies [20], pre-setting an alpha error of 5%, meaning that a significant difference will be present with 95% confidence.

It was estimated that, for this study, *n* = 58.40. Therefore, 60 subjects were selected and divided into two groups.

### 2.2. Sample Selection

Following the study approval by the Ethics Committee of Santiago de Compostela-Lugo, Spain (Process no. 2023/444), 60 patients were selected from those who attended our department and required a direct Class II restoration. Written informed consent was obtained from all subjects. The cases were chosen based on the following criteria: adults (≥18 years), presence of a Class II carious lesion regardless of pulp involvement in posterior teeth, natural tooth adjacent to the lesion (intact or with an adequate approximal restoration according to FDI criteria) [21,22], no diastemas, no need for indirect restoration, and no need for crown lengthening or similar techniques. Two calibrated evaluators checked the inclusion and exclusion criteria of the participants. The evaluations were performed by clinical examination and by analyzing the bite-wing radiographies (Carestream Dental, Assago, Italy) made with XCP Rinn (Rinn, Weld, CO, USA).

Patients included in the study were randomized into two groups: Group 0 (Conventional technique) and Group 1 (Digital technique). Block randomization was used to ensure equality of the groups using Macro !RNDSEQ V2011.09.09 [23].

### 2.3. Digital Technique Description

This new technique has two phases: (1) Measurement of the wedges; (2) Selection of the wedge.

#### 2.3.1. Measurement of the Wedges

Different wedges from different brands were selected and measured.

- Composi-Tight 3D Fusion Wedge (Garrison, Spring Lake, MI, USA).
- WedgeWand (Garrison, Spring Lake, MI, USA).
- Hawe Sycamore Interdental Wedges (KerrHawe, Bioggio, Switzerland).
- BioClear Biofit HD Posterior Wedge (Bioclear, Tacoma, WA, USA).
- Palodent Plus (Dentsply Sirona, Konstanz, Germany).

For the wedge measurement, a digital caliber (Mitutoyo Corporation, Kawasaki, Japan) was used. At the midpoint of the wedge in the longitudinal direction, a vertical line was made with a fine-tip indelible marker (Staedtler Mars GmbH & Co. KG, Nuremberg, Germany). At this point, the wedges were measured in height and thickness (Table 1 and Table 2) because, ideally, the wedge should be positioned so that this point is located in the middle of the contact area in a mesio-distal direction. For Palodent Plus (Dentsply Sirona, Konstanz, Germany), BioClear Biofit HD Posterior Wedge (Bioclear, Tacoma, WA, USA), Composi-Tight 3D Fusion Wedge, and WedgeWand (Garrison, Spring Lake, MI, USA) measurements, it was subtracted from the total length the end by which the wedge is held.

#### 2.3.2. Selection of the Wedge

(a) Radiographic measurement.

**Figure 1:**
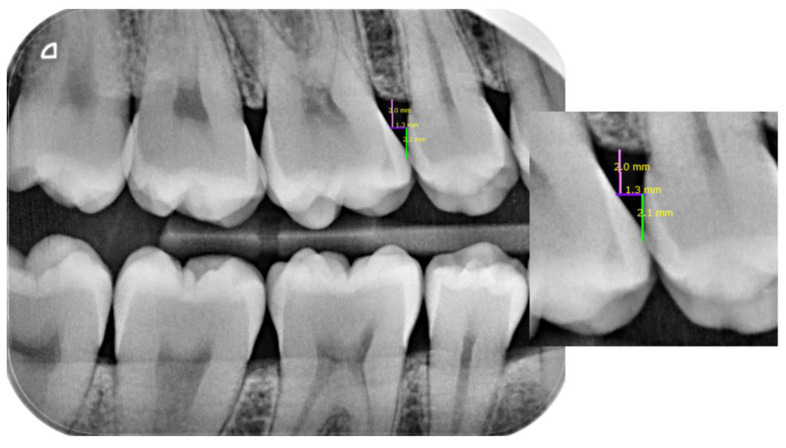
Measurements on radiography with color lines. Pink line: 2 mm height from the bone crest. Purple line: cervical embrasure measurement in mesio-distal direction at 2 mm from the bone crest (width of the wedge). Green line: distance between the ideal contact area to 2 mm from the bone crest (height of the wedge).

For the radiographic measurement of the cervical embrasure, both height and width must be considered. As previously reported, a direct composite restoration could be placed in a predictable manner and without inflammatory problems at 2 mm from the bone crest as adequate space for connective tissue attachment was maintained. If the distance is lower, a surgical crown lengthening or similar technique is indicated to give this space to the connective tissue. Based on these findings, a line 2 mm from the bone crest was established as the limit for measuring the interproximal space [24]. The width is determined by the space between the approximal surfaces of the teeth in the mesio-distal direction at 2 mm from the bone crest. The wedge should always have a width equal to the interproximal space or slightly wider, as previously described, in order to achieve a wedge effect and stable positioning [15]. The height corresponds to the distance resulting from the point where the contact area would be ideally located [10,11] up to 2 mm from the bone crest (Figure 1).

(b) Suggested wedge according to the cervical embrasure measurements.

Based on the sizes of the wedges and according to the width of the cervical embrasures, a series of 3 intervals in ascending order was established. Once the measurements on the radiography are complete, the wedge selection procedure is carried out by choosing the one that fits as close as possible to the provided dimensions. Thus, from all the wedges of the chosen interval, the one that fits better according to the measured height on the radiography must be selected. When encountering situations where available wedges do not precisely fit the measurements, adjustments can be made, emphasizing the constant nature of the cervical embrasure.

First interval: 0.89 to 1.39 mm width embrasures.
  - Garrison WedgeWand X-Small/yellow.
  - Bioclear Biofit Small/pink.
  - Garrison Composi-Tight 3D Fusion Wedge yellow.
  - Palodent Plus Small/dark blue.
  - Garrison Composi-Tight 3D Fusion Wedge orange.
  - Bioclear Biofit Medium/orange.
  - Palodent Plus Medium/blue.
  - Garrison Composi-Tight 3D Fusion Wedge green.
  - Garrison WedgeWand Small/blue.
  - Hawe Sycamore Interdental Wedges orange.

Second interval: 1.40 to 1.89 mm width embrasures:
  - Bioclear Biofit Deep Caries/green.
  - Garrison WedgeWand Medium/orange.
  - Hawe Sycamore Interdental Wedges white.
  - Bioclear Biofit Large/yellow.
  - Hawe Sycamore Interdental Wedges green.
  - Palodent Plus Large/light blue.
  - Hawe Sycamore Interdental Wedges blue.
  - Bioclear Biofit Extra Large/blue.

Third interval: 1.90 to 2.39 mm width embrasures:
  - Hawe Sycamore Interdental Wedges yellow.
  - Garrison WedgeWand Large/green.
  - Hawe Sycamore Interdental Wedges pink.

### 2.4. Treatment Protocol

All the treatments were performed by first-year students studying for a Master’s Degree in Advanced Endodontics, Restorative, and Esthetic Dentistry (University of Santiago de Compostela, Spain), under supervision of experienced clinicians. Half of the students were trained in the new preoperative wedge selection method, while the other half performed the conventional wedge selection technique as taught at undergraduate level. Subsequently, according to the randomization sequence:

In Group 0 (*n* = 30), once local anesthesia was applied, isolation was achieved with rubber-dam (Dental Dam, Nic Tone, MDC Dental; Zapopan, Mexico). Next, carious tissue and unsupported enamel were removed with high-speed diamond burs and contra-angle tungsten carbide burs under water irrigation. After the space evaluation, a pre-contoured sectional matrix (Palodent Matrix System, Dentsply Caulk, Milford, DE, USA, thickness: 38 μm) was selected, taking into account the criteria mentioned in previous protocols [7], and placed with the wedge that the operator considered appropriate through visual evaluation [12], from one of the following brands: Composi-Tight 3D Fusion Wedge (Garrison, Spring Lake, MI, USA), WedgeWand (Garrison, Spring Lake, MI, USA), Hawe Sycamore Interdental Wedges (KerrHawe, Bioggio, Switzerland), BioClear Biofit HD Posterior Wedge (Bioclear, Tacoma, WA, USA), and Palodent Plus (Dentsply Sirona, Konstanz, Germany), along with a separation ring (Dentsply Sirona, Konstanz, Germany). In case of poor adaptation, the wedge was replaced by another one. After matrix stabilization, a universal adhesive (3M™ Scotchbond™ Universal Plus, 3M ESPE, Seefeld, Germany) was applied under selective enamel etching. A small particle hybrid composite (Grandio; Voco, Cuxhaven, Germany) was placed following the incremental technique (1.5–2 mm thick layers) in order to reduce the polymerization shrinkage stress. Finally, occlusion verification and polishing were performed. The quality of the treatment was tested by clinical and radiographic examination.

**Figure 2:**
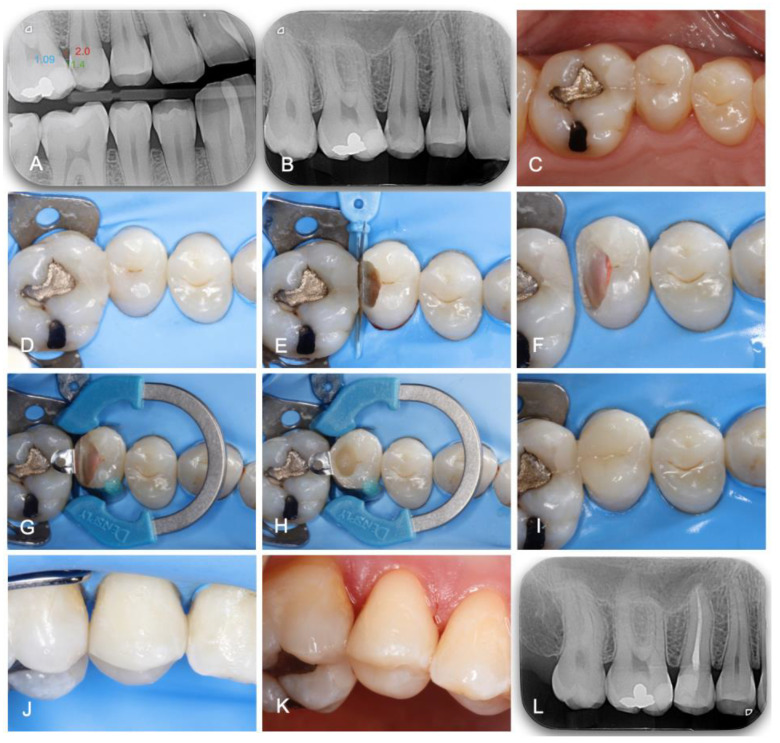
Clinical case where both endodontic and restorative procedures were performed in one appointment. (**A**) Bite-wing radiography and embrasure measurement. (**B**) Preoperative periapical radiography. (**C**) Preoperative view. (**D**) Isolation. (**E**) Pre-wedging and adjacent tooth protection. (**F**) Root canal treatment. (**G**) Placement and stabilization of the matrix. (**H**) Class II transformation into Class I. (**I**–**K**) Final view. (**L**) Final bite-wing radiography.

In Group 1 (*n* = 30), the treatment was performed using the digital method. On the preoperative bite-wing radiography, the appropriate measurements were performed as described above. According to the obtained results, the best adapted wedge was selected from the different brands used in the study. This was conducted preoperatively, after the patient’s first visit/review. The restorative procedure per se was performed in the same way as in Group 0, as shown in Figure 2. After local anesthesia, the operative field was isolated under rubber-dam (Dental Dam, Nic Tone, MDC Dental; Zapopan, Mexico). High-speed diamond burs and contra-angle tungsten carbide burs were used for caries removal. Then, a pre-contoured sectional matrix (Palodent Matrix System, Dentsply Caulk, Milford, DE, USA, thickness: 38 μm), with the previously selected wedge and a separation clamp (Dentsply Sirona, Konstanz, Germany) were placed. In case of poor fitting, as in Group 0, the wedge was replaced. A universal adhesive (3M™ Scotchbond™ Universal Plus, 3M ESPE, Seefeld, Germany) was applied under selective enamel etching. The nano-hybrid composite (Grandio; Voco, Cuxhaven, Germany) was applied in a multilayering technique. After the occlusal analysis and final polishing, the quality of the treatment was checked by clinical and radiographic inspection.

### 2.5. Evaluation of Class II Direct Restorations

In both groups, the number of wedges used during matrix placement and stabilization was registered, along with their modifications for achieving better adaptation. Two calibrated and blinded evaluators recorded the following data. A visual inspection was made to determine the presence of wedge damage to the adjacent tissues. According to FDI criteria scores 1 and 2, the contact area was considered correct when it had a normal contact point, where dental floss or a 25 µm metal blade could pass or when contact was slightly too strong but dental floss or a 25 µm metal blade could pass with pressure, and the contour was also normal or slightly deficient [21]. It was considered incorrect when the contact was weak and a ≥50 µm metal blade could pass and the contour was inadequate [21]. Marginal adaptation was considered successful when the contour was harmonious, without gaps, steps, or the presence of discolored lines or when these could be corrected by polishing, while it was considered unsuccessful when the gap was ≥150 µm or the defects could not be solved easily [21]. For gap measurement, a special probe (Deppeler, Rolle, Switzerland) with a tip diameter of 150 µm was used, as reported previously [21]. When the final result of the restorations was unsuccessful, repair or replacement of them was carried out [21,22].

### 2.6. Statistical Analysis

The intraclass correlation coefficient was used to estimate the reliability of the measurements taken by two evaluators. The Kolmogorov–Smirnov test was applied to examine the distribution of the data. Student’s *t*-test was used for the quantitative variable and Pearson Chi-square and Fisher’s exact tests were used for categorical ones. SPSS Version 29.0 (IBM Corp, Armonk, NY, USA) was used for statistical analysis. The cut-off for statistical significance was determined to be *p* < 0.05.

## 3. Results

The inter-examiner reliability score for the risk of bias between the evaluators was 0.96, showing that the agreement was very high. For the variable number of used wedges (Table 3), the mean and SD values were 1.50 ± 0.50 in Group 0 (Conventional method) and 1.00 ± 0.00 in Group 1 (Digital technique). Due to the normal distribution of the data (Kolmogorov–Smirnov test, *p* > 0.05), Student’s *t*-test was used to compare the means of both groups, where *p* = <0.001 was statistically significant. With 95% confidence interval, the number of used wedges was 0.31 to 0.69 fewer in Group 1 than in Group 0.

Table 4 shows the binary categorical variables. The Pearson Chi-Square test was used for wedge modifications, tissue laceration, and contact area, while Fisher’s exact test was used for the marginal adaptation variable, because the assumption of expected frequencies was not met. For wedge modifications, *p* = 0.774, so there are no statistically significant differences between the groups. However, the prevalence of modifications is lower in Group 1 (26.70%) than in Group 0 (30.00%). Concerning tissue laceration, *p* =< 0.001, so there are statistically significant differences between groups, with a lower prevalence in Group 1 (10.00%) compared to Group 0 (53.30%). *p* =< 0.001 for contact area; therefore, there are statistically significant differences among both groups, being better in Group 1 (100.00%) than in Group 0 (63.30%). For marginal adaptation, *p* = 0.024, showing statistically significant differences between groups, with a better adaptation in Group 1 (100.00%) than in Group 0 (80.00%).

## 4. Discussion

Restoring the approximal anatomy in a Class II direct restoration presents a significant challenge in restorative dentistry [25]. When this anatomy is not accurately reconstructed, adverse outcomes such as masticatory discomfort, caries, periodontal problems, and undesired movement of teeth may arise; restoration of the approximal anatomy is a big challenge [7]. Prefabricated sectional matrices are considered more effective than circumferential ones for reproducing the contact area and provide superior contact tightness [1,26]. However, both matrix systems exhibit deficiencies in accurately recreating the interproximal anatomy [1,26]. The preference for circumferential matrices by many dentists, despite the perceived advantages of sectional matrices, is attributed to the latter being more technique-sensitive, which can present a significant barrier, particularly for those clinicians with less experience [14]. Therefore, it is very important to develop new techniques and protocols to facilitate their placement and stabilization.

A requirement for Class II restoration is to reproduce the morphology of an intact natural tooth. Therefore, it is important to select a matrix with a proper height [7]. The matrix band should be positioned ±0.5 mm above the marginal ridge of the adjacent tooth to obtain a correct contour in the cervico-occlusal direction [7]. The maximum curvature of the matrix must also be considered [7]. A greater distance between the cervical cavity margin and the adjacent tooth will determine an increase in the maximum curvature of the matrix [7]. Criteria for proper matrix selection have been published [7,14]. However, until now there are no specific protocols for the correct selection of the elements used to stabilize and adapt the matrix.

Stabilization of the matrix band can be obtained using a wedge, a separation ring, teflon tape, and block-out or flowable resin [7]. Among these, wedges are the most-used tool for achieving cervical sealing and matrix stabilization [14]. Although some manufacturers recommend evaluating visually the preoperative bite-wing radiography in order to select the appropriate wedge, at the moment there are no published guides or techniques that could be used by the clinicians to choose it. Instead, the wedge is selected visually according to the size that the clinician believes to be approximately correct, often having to try several dimensions. In addition to the loss of working time, the risk of tissue damage increases. Preoperative wedge selection is a minimal invasive technique according to the fact that the interdental tissue is not damaged by testing different wedges sizes; furthermore, it reduces operative time and wedge wasting, as shown in the results of the study.

There are wooden, plastic, and silicone manufactured wedges. Some authors prefer hard wooden wedges more than soft ones, as they exert greater force to separate [7]. On the other hand, the flexibility of plastic and silicone wedges may provide better cervical adaptation [7,14] and seem to be less invasive for soft tissues, although efficiency in force exerting is reduced [7]. As mentioned in the introduction section, the correct height and width of the wedge will be crucial to avoid undesirable effects. As shown in the study, the presence of poor marginal adaptation and an inadequate contact area were statistically more frequent in Group 0. In many cases, it will be necessary to customize the wedge to achieve the desirable objectives, modifying them with the help of a bur, for example [14,27]. In this study, the modifications weren’t statistically significant between the two groups, and this is probably due to the limited number of wedge heights and widths available on the market.

This new selection method will be particularly useful in cases where visibility is compromised, such as in cavities with deep margins, where assessing the suitability of the wedge is difficult. When the remaining tooth structure is located at the level of the gingival sulcus or epithelium, it is necessary to perform a deep marginal elevation [28]. If this is not possible and the cavity reaches the connective tissue or the bone crest, it will be necessary to perform another technique, such as crown lengthening [28,29,30]. Traditionally, a distance of 3 mm from the preparation margin to the bone was recommended [8]. However, according to an in vivo study carried out in 2018 in dogs, these techniques can be performed predictably if the preparation margin is located 2 mm from the bone crest, as long as the adaptation and polishing of the material is optimal [24]. For this reason, 2 mm from the bone crest is taken as a reference for the measurements in this study.

Bite-wing radiography is the recommended projection for proximal caries detection [31,32], and the utilization of image receptor holders enhances the technique’s efficacy, as they ensure that the film is placed parallel to the tooth axis and perpendicular to the X-ray beam [33], minimizing overlapping effects on proximal surfaces and ensuring comparability between different images of the same teeth for caries monitoring [32]. Beyond allowing the correct diagnosis of proximal caries, bite-wing technique and radiological positioners are required for performing the present technique. By avoiding the shortening or lengthening of the image and enhancing the visualization of the interproximal area, the measurement accuracy required is assured. Furthermore, the use of digital radiography is mandatory. Apart from being more efficient than conventional radiography [34], it provides additional options in digital processing, such as the image measurement, that is required for performing this technique.

The proposed preoperative wedge selection has some limitations and should be improved by extending the measurements to a larger variety of commercialized wedges in order to provide a guide that any clinician could consult. Nevertheless, due to the manufacturing process, wedges could not always present the performed sizes. As the precise embrasure measurement is key for this technique, it can succeed only if bite-wing radiography and radiological positioners are used. On the other hand, teeth malpositions, previous restorations with incorrect approximal anatomy, periodontal disease, or bone crest defects can affect the radiographic assessment of the cervical embrasure. For this reason, each case has to be individualized.

## 5. Conclusions

Within the limitations of this current study, it was concluded that this technique gives an accurate and reproducible way to preoperatively select the wedge for Class II direct restorations through the measurement of cervical embrasures on the bite-wing radiography. It reduces wedge wasting and provides a minimal invasive approach for interdental tissue. Furthermore, it leads to achieving a correct marginal adaptation and thus accurately reproduce the contact area, and it can be especially useful for inexperienced operators or dental students.

## Figures and Tables

**Table 1:** Measurement of the wooden wedges in height and width at the level of the midpoint in the longitudinal direction.

Wedge (Trade Name)	Model/Colour	Width (mm)	Height (mm)
Hawe Sycamore Interdental Wedges (KerrHawe, Bioggio, Switzerland)	Orange	1.22	1.81
	White	1.66	1.85
	Green	1.71	2.01
	Blue	1.81	2.08
	Yellow	1.91	2.00
	Pink	2.39	2.65

**Table 2:** Measurement of the plastic wedges in height and width at the level of the midpoint in the longitudinal direction.

Wedge (Trade Name)	Model/Colour	Width (mm)	Height (mm)
Garrison Composi-Tight® 3D Fusion™ Wedge (Garrison, Spring Lake, MI, USA)	Yellow	1.00	1.55
	Blue	1.10	1.66
	Orange	1.11	1.99
	Green	1.16	1.97
Garrison WedgeWand (Garrison, Spring Lake, MI, USA)	X-Small/yellow	0.89	1.11
	Small/blue	1.21	1.49
	Medium/orange	1.64	2.06
	Large/green	2.06	2.31
BioClear Biofit HD Posterior Wedge (Bioclear, Tacoma, WA, USA)	Small/pink	0.90	1.40
	Medium/orange	1.11	1.49
	Deep Caries/green	1.50	1.73
	Large/yellow	1.67	1.75
	Extra Large/blue	2.11	1.84
Palodent Plus (Dentsply Sirona, Konstanz, Germany)	Small/dark blue	1.01	1.50
	Medium/blue	1.16	1.97
	Large/light blue	1.75	1.85

**Table 3:** Descriptive analysis of the wedges used to perform restorative procedures.

Group	Mean	SD	*p* Value	95% Confidence Interval
*0 (Conventional technique) n = 30*	1.50	0.50	<0.001	−0.690;−0.310
*1 (Digital technique) n = 30*	1.00	0.00		

**Table 4:** Number of restorations with deficiencies, tissue damage, and/or wedge modifications.

Group	Deficient Contact Area	Poor Marginal Adaptation	Tissue Laceration	Wedge Modifications
*0 (Conventional technique) n = 30*	11 ^a^	6 ^a^	16 ^a^	9
*1 (Digital technique)* *n = 30*	0 ^b^	0 ^b^	3 ^b^	8

^a,b^ Statistically significant differences between groups (*p* < 0.05).

## Data Availability

The original contributions presented in the study are included in the article, further inquiries can be directed to the corresponding author.
